# Tree species, tree genotypes and tree genotypic diversity levels affect microbe-mediated soil ecosystem functions in a subtropical forest

**DOI:** 10.1038/srep36672

**Published:** 2016-11-18

**Authors:** Witoon Purahong, Walter Durka, Markus Fischer, Sven Dommert, Ricardo Schöps, François Buscot, Tesfaye Wubet

**Affiliations:** 1 UFZ-Helmholtz Centre for Environmental Research, Department of Soil Ecology, Theodor-Lieser-Str. 4, D-06120 Halle (Saale), Germany; 2 UFZ-Helmholtz Centre for Environmental Research, Department of Community Ecology, Theodor-Lieser-Str. 4, D-06120 Halle (Saale), Germany; 3 German Centre for Integrative Biodiversity Research (iDiv), Halle-Jena-Leipzig, Deutscher Platz 5e, D-04103 Leipzig, Germany; 4 Institute of Plant Sciences, University of Bern, Altenbergrain 21, 3013 Bern, Switzerland

## Abstract

Tree species identity and tree genotypes contribute to the shaping of soil microbial communities. However, knowledge about how these two factors influence soil ecosystem functions is still lacking. Furthermore, in forest ecosystems tree genotypes co-occur and interact with each other, thus the effects of tree genotypic diversity on soil ecosystem functions merit attention. Here we investigated the effects of tree species, tree genotypes and genotypic diversity levels, alongside soil physicochemical properties, on the overall and specific soil enzyme activity patterns. Our results indicate that tree species identity, tree genotypes and genotypic diversity level have significant influences on overall and specific soil enzyme activity patterns. These three factors influence soil enzyme patterns partly through effects on soil physicochemical properties and substrate quality. Variance partitioning showed that tree species identity, genotypic diversity level, pH and water content all together explained ~30% variations in the overall patterns of soil enzymes. However, we also found that the responses of soil ecosystem functions to tree genotypes and genotypic diversity are complex, being dependent on tree species identity and controlled by multiple factors. Our study highlights the important of inter- and intra-specific variations in tree species in shaping soil ecosystem functions in a subtropical forest.

Individual tree genotypes and genotypic diversity play crucial roles in shaping the structure of communities of organisms in forests by mediating covariance among various associated functional groups such as microorganisms, lichens and invertebrates^1^. As have been shown in the cases of *Populus angustifolia* and *Populus balsamifera*, different tree genotypes can support specific soil microbe, leaf pathogen, foliar fungi, twig endophyte, lichen and arthropod communities^2^,^3^,^4^,^5^,^6^,^7^. Such tree genotype-specific organism communities have been reported to be heritable and consistent across different years^4^. However, the extent of this community-shaping effect mediated by tree genotype varies according to the species of tree, the environmental conditions, and the associated functional group under consideration, as was shown for the root-microbiome of different genotypes of *Populus deltoides*^8^. Most previous studies have analyzed the effects of the genotypes of individual trees on the composition of dependent communities; it is still unknown how tree genotypes and genotypic diversity affect ecosystem functions^1^,^2^,^3^,^7^.

Measuring enzyme activities in the soil remains the most direct way to investigate soil ecosystem functions^9^,^10^, and soil enzyme activities are often used as indicators of soil health and stability^11^,^12^,^13^. In soils, microorganisms produce various enzymes that decompose plant litter and organic compounds; however changes in the structure of the microbial community are not always linked with changes in enzyme activities and the associated ecosystem functions^10^. This uncoupling between structure and function can be explained by both functional redundancies within microbial communities and the absence of any quantitative link between microbial growth and functions^9^,^10^.

Enzyme activities in forest soil are affected by both biotic and abiotic factors^14^. Among the biotic factors, the composition of tree species, tree species identity and microbial community have been found to be significant predictors of soil enzyme activities^15^,^16^,^17^,^18^,^19^. Of the abiotic factors, soil physicochemical properties and substrate quality have often been reported as significantly affecting soil enzyme activities^14^,^16^. Biotic and abiotic factors in forest soils are closely linked to the identity of the dominant tree species (interspecific variation), which occupy a pivotal function in forest ecosystems 16,17,20. In particular, feedback between the roots of dominant trees and their associated soil microbial community governs soil enzyme activities^21^ and consequently has a major impact on ecosystem functions^16^,^21^. In addition to tree species identity and diversity, genetic variation within a tree species (intraspecific variation), i.e. the identity^22^,^23^ and diversity^24^ of tree genotypes, has been shown to significantly influence growth, performance and the responses of trees to different environmental parameters. Because of this, factors influenced by tree genotype, e.g. litter quality and root exudate profiles, are also expected to impact on the rhizosphere microbial community and the associated enzymatic activities^22^.

Soil is the largest pool of organic carbon in terrestrial ecosystem^25^,^26^. Organic carbon as well as other organic compounds are distributed to the soil through plant litter and root exudates^13^. These organic compounds consist of both readily available substrates such as sugars, starch, organic and amino acids, and larger complex substrates such as cellulose, hemi-cellulose and lignin which can be decomposed and transformed to low-molecular-mass compounds by a variety of microbial extracellular enzymes in the soil^13^,^27^,^28^. The low-molecular-mass compounds can then be available in soil for microbial and plant assimilations^29^. In this context, soil extracellular enzymes produced by microorganisms play vital roles in the biogeochemical reactions of organic matter decomposition, regulate global carbon and nutrient cycles and thus maintain soil functions in terrestrial ecosystem^30^. Extracellular soil activities are reported to be related to amounts of organic matter inputs, organic carbon, total nitrogen and microbial activity. For examples low and high amounts of leaf litter in the study forest plots show tendencies to decrease and increase enzyme activities (and also organic carbon, total nitrogen and microbial activity), respectively^31^,^32^. These shifts in enzyme activity patterns could thus, reflect changes in relative nutrient limitations with altered organic matter inputs as well as indicate the nutrient status and soil microbial activity of the study area^31^,^32^.

Although soil enzyme activities are useful indicator for soil health and quality, there are some limitations and cautions that should be considered^30^. These include, (i) inability of currently used enzyme assays to distinguish between the contributions of different fractions of enzyme activities (i.e. enzymes absorbed by inorganic or organic colloids, enzyme activities due to abiotic transformation and intracellular enzymes in viable cells), (ii) to investigate the microbial functional diversity, measuring a range of enzymes that reflect the same soil ecosystem function of interest is needed otherwise, it is important to identify and measure the key enzyme catalyzing the rate-limiting step of the overall metabolic pathway, (iii) results from specific and overall pattern (i.e. by means of multivariate analysis) of enzyme activities are useful information as the result from overall pattern alone may hinder the specific enzyme activity, and (iv) enzyme activities are highly variable across space and time^13^,^30^.

Research has generally focused on enzymes important for the acquisition of macronutrients such as carbon (C), nitrogen (N) and phosphorus (P)^9^,^10^,^16^,^33^. In this study we used β-glucosidase and xylosidase to represent enzymes of C acquisition^10^. β-glucosidase catalyzes the hydrolysis of glycosidic bonds in β-D-glucosides and oligosaccharides, whereas xylosidase is important for the degradation of xylooligomers into xylose^27^. N-acetylglucosaminidase (NAG) and acid phosphatase are representative of N and P acquisition respectively^10^. NAG catalyzes the hydrolysis of N-acetyl-beta-D-glucosaminide residues in chitin-derived oligomers, while acid phosphatase catalyzes the hydrolysis of phosphoric (mono) ester bonds (i.e. it mineralizes organic phosphorus into phosphate) under acidic conditions^27^. In addition, we investigated two oxidative enzymes (phenol oxidase and general peroxidase) important in lignin degradation and humus formation, which are important processes in soil C stabilization^15^.

In this study we carried out an experiment in a recently established platform on genetic diversity in forest trees within the Biodiversity-Ecosystem Functioning (BEF) China project^34^. Given the fact that the contribution made by tree genotypes to soil microbial communities and their enzymatic activity may depend on the identity of the tree species concerned, we investigated soil enzyme profiles in the root zone of different genotypes of four broadleaf tree species (*Alniphyllum fortunei*, *Cinnamomum camphora*, *Daphniphyllum oldhamii* and *Idesia polycarpa*) planted at different levels of genetic diversity (Tables S1 and S2).

The major objectives of this study were to investigate (i) the effects of tree species identity, tree genotype and genotypic diversity level on the overall patterns of soil enzyme activity, and (ii) the specific responses of different enzymatic activities important for C, N and P cycling and acquisition to tree species identity, tree genotype and genotypic diversity level. We hypothesized that tree species identity, tree genotype, and genotypic diversity have significant effects on the overall and specific patterns of soil enzyme activity. We assume that interspecific variation has a greater influence on soil physicochemical properties compared to the intraspecific variation. As the four tree species (as compared to the four tree genotypes) used in this experiment differ greatly in leaf litter chemical composition, we expect tree species identity to have more influence than tree genotypes on patterns of soil enzyme activity. Due to plant-microbe and microbe-microbe interactions we hypothesized that there is less competition among soil microbes in a mono-genotypic treatment compared with a multi-genotypic treatment^35^ and thus, we expected higher enzyme activities in the mono-genotypic treatment.

## Results

### Overall patterns of soil enzyme activities: interplay between tree species identity, genotype and genotypic diversity level

**Figure 1:**
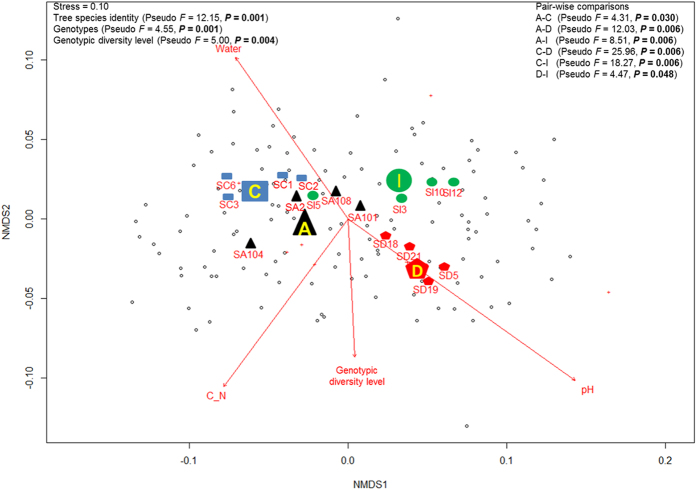
Non-metric multidimensional scaling (NMDS) ordination of activities of six soil enzymes under four tree species. Significant factors (*P* < 0.05) correlated with overall patterns of enzyme activities are shown. C_N = soil C: N ratio; Water = soil water content. The effects of tree species identity (A = *Alniphyllum fortunei*; C = *Cinnamomum camphora*; D = *Daphniphyllum oldhamii* and I = *Idesia polycarpa*), tree genotypes (S = seed family) and genotypic diversity analyzed using PERMANOVA are shown on the top left. Pair-wise comparisons of overall patterns of enzyme activities between different tree species are shown on the top right.

**Figure 2:**
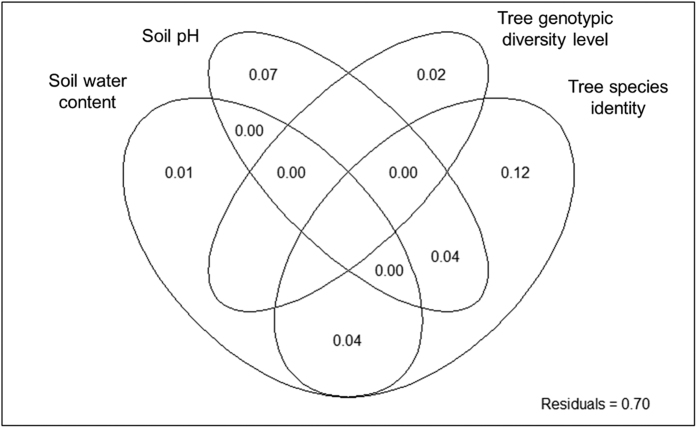
Variance partitioning analysis to determine how plant and soil related factors explain variance in enzyme activity pattern. Each circle represents the portion of variation accounted for each factor. Shared variance is shown in the intersecting portions of the circles.

Tree species identities, tree genotype and genotypic diversity significantly affected the overall patterns of soil enzyme activity (*P* < 0.01; Fig. 1). They were also correlated with soil physicochemical properties (water content, pH) and substrate quality (C:N ratio) (Fig. 1; Table 1). Distance-based redundancy analysis (dbRDA) showed that tree species identity, genotypic diversity level, soil pH and soil water content were most influential for the overall patterns of soil enzymes (*P* < 0.05) and were retained for variance partitioning analysis (Table 2).Variance partitioning showed that enzyme activities were explained by plant (tree species identity = 12%; genotypic diversity level = 2%) and soil (pH = 7%; water content = 1%) related factors and the shared fraction between these factors account for 8% (tree species identity and pH = 4%; tree species identity and water content = 4%) (Fig. 2). Pairwise comparisons among tree species showed that the overall enzyme patterns associated with different tree species were significantly different (*P* = 0.048–0.006; Fig. 1).

**Figure 3:**
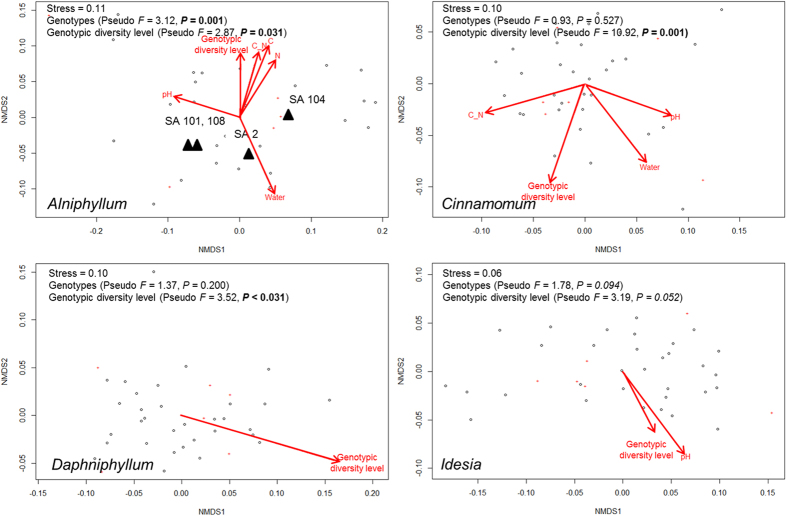
Non-metric multidimensional scaling (NMDS) ordination of activities of six soil enzymes in each of four tree species. Significant factors (*P* < 0.05) correlated with overall patterns of enzyme activities are shown. C_N = soil C: N ratio; Water = soil water content. The effects of tree genotypes (S = seed family) and genotypic diversity analyzed using PERMANOVA are shown on the top left.

In each of the four tree species, overall patterns of soil enzyme activities responded differently to genotype and soil physicochemical and substrate quality. Only genotypic diversity level significantly correlated with the observed patterns of soil enzymes across all tree species (Table 1). Soil pH significantly correlated with the pattern of soil enzymes in three out of the four species (Table 1). For *Alniphyllum*, level of genotypic diversity, together with tree genotype, soil physicochemical properties (water content, pH) and substrate quality (C content, N content, C: N ratio) shaped the overall enzyme pattern (Table 1, Fig. 3). The effect of tree genotype was not significant for the other three species. In *Cinnamomum*, enzyme patterns correlated with genotypic diversity level and soil physicochemical properties (water content, pH) and substrate quality (C: N ratio) (Table 1, Fig. 3). In *Daphniphyllum*, however, the pattern was correlated solely with genotypic diversity level and in *Idesia* there were correlations with genotypic diversity level and soil pH (Table 1, Fig. 3). Permutational Multivariate Analysis of Variance (PERMANOVA) confirmed that tree genotype significantly affected overall soil enzyme activities in *Alniphyllum,* while genotypic diversity level played a significant role in *Alniphyllum*, *Cinnamomum* and *Daphniphyllum* but its effect was only marginally significant in *Idesia (P* = 0.052; Fig. 3).

### Effect of tree species identity on specific enzyme activities

**Figure 4:**
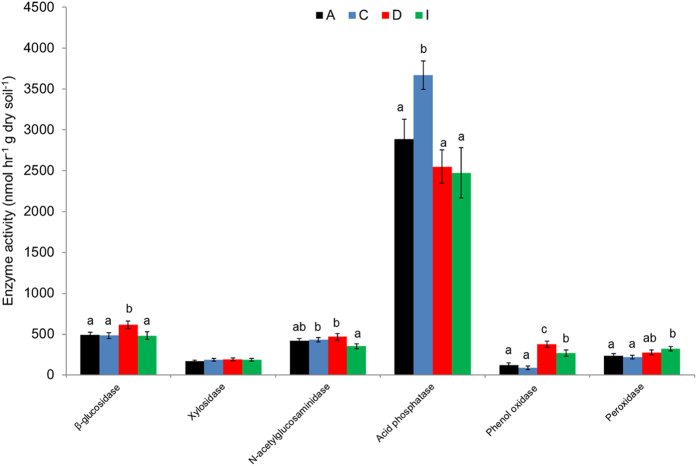
Enzyme activities (mean ± SE) in soil samples collected under four different tree species (A = *Alniphyllum fortunei*; C = *Cinnamomum camphora*; D = *Daphniphyllum oldhamii* and I = *Idesia polycarpa*). Different letters indicate significant differences (*P* < 0.05) between species according to split-plot ANOVA and an LSD test.

Tree species identity significantly influenced most of the enzyme activities measured, the exception being xylosidase (Fig. 4). The activities of the two hydrolytic enzymes related to C acquisition responded differently to tree species identity. β-glucosidase activity was highest in soil close to *Daphniphyllum* and lower for the other three tree species, while xylosidase activity was similar for all four species (*P* > 0.05). NAG activity was highest for *Cinnamomum* and *Daphniphyllum* and lowest for *Idesia*. Acid phosphatase activity was (27–48%) higher in *Cinnamomum* than in the other three tree species. Phenol oxidase activity was highest for *Daphniphyllum* followed by *Idesia* and lowest for *Alniphyllum* and *Cinnamomum*. Peroxidase activity was also lowest in the case of *Alniphyllum* and *Cinnamomum* and highest in *Idesia*. Taking the overall results for all specific enzyme activities, we found that different tree species significantly impact on C, N and P related enzymes in different ways.

### Responses of specific enzymes to genotype and genotypic diversity levels of the different tree species

**Figure 5:**
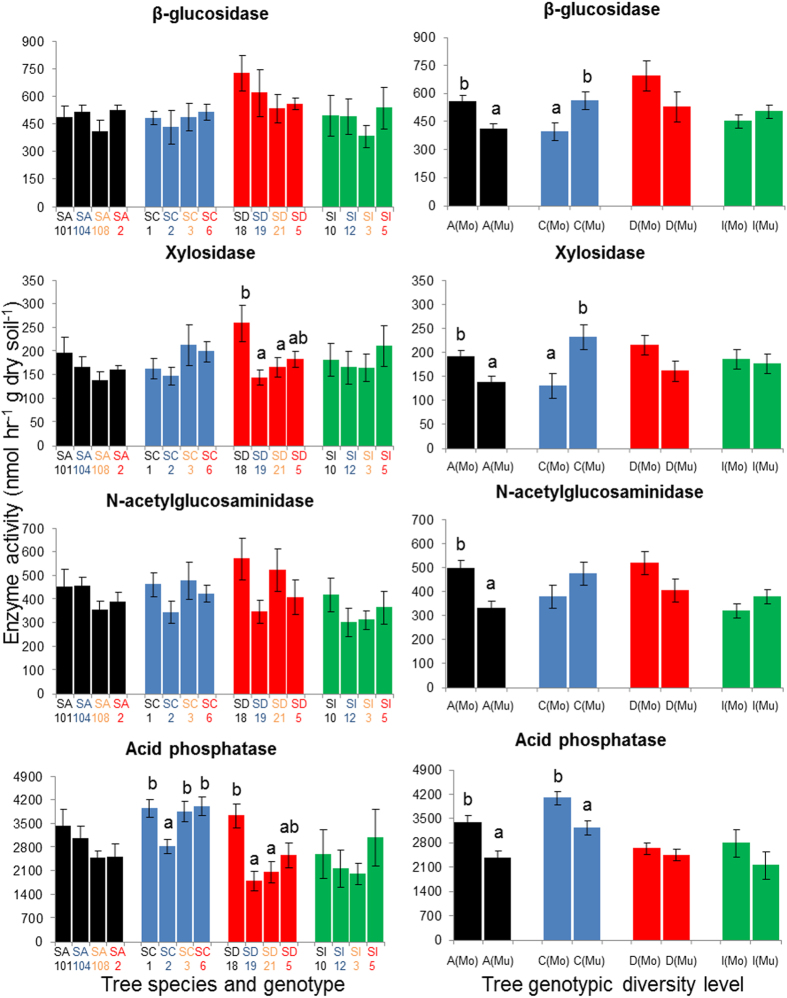
Hydrolytic enzyme activities (mean ± SE) in soil samples collected under four different genotypes (SA = seed family from *Alniphyllum fortune* (**A**); SC = seed family from *Cinnamomum camphora* (**C**); SD = seed family from *Daphniphyllum oldhamii* (**D**) and SI = seed family from *Idesia polycarpa* (**D**)) and two genotypic diversity levels (Mo = mono-genotypic treatment; Mu = multi-genotypic treatment). Different letters indicate significant differences (*P* < 0.05) between different genotypes or genotypic diversity levels according to split-plot ANOVA and an LSD test (the latter only for different genotypes).

**Figure 6:**
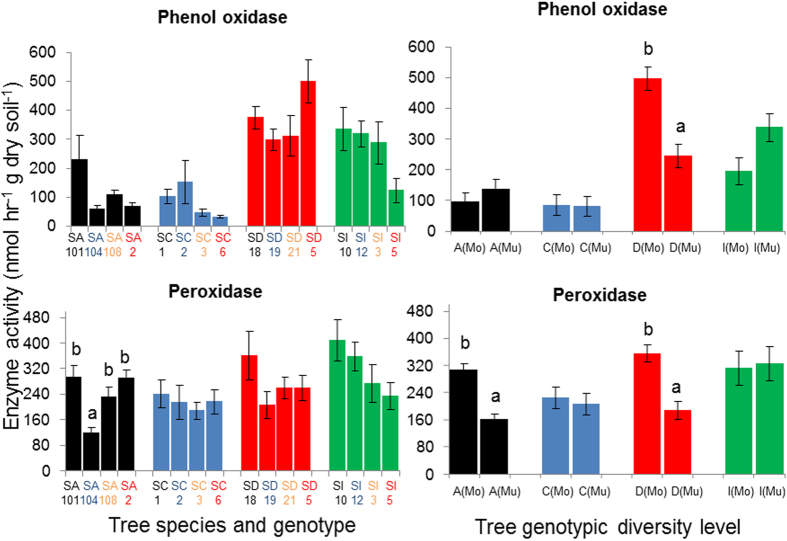
Oxidative enzyme activities (mean ± SE) in soil samples collected under four different genotypes (SA = seed family from *Alniphyllum fortune* (**A**); SC = seed family from *Cinnamomum camphora* (**C**); SD = seed family from *Daphniphyllum oldhamii* (**D**) and SI = seed family from *Idesia polycarpa* (I)) and two genotypic diversity levels (Mo = mono-genotypic treatment; Mu = multi-genotypic treatment). Different letters indicate significant differences (*P* < 0.05) between different genotypes or genotypic diversity levels according to split-plot ANOVA and LSD test (the latter only for different genotypes).

Responses of the different soil enzyme activities to tree genotype and genotypic diversity level varied among the four tree species. In all but one species, tree genotype and genotypic diversity level showed significant effects on some enzymes (Figs 5 and 6). In *Idesia*, however, none of the six soil enzymes measured responded to tree genotype or genetic diversity (*P* > 0.05; Figs 5 and 6).

In *Alniphyllum*, tree genotypic diversity level had greater effects than tree genotype, and it affected the activity of all enzymes except phenol oxidase (Figs 5 and 6). Multi-genotypic treatment resulted in lower enzyme activities than mono-genotypic treatment in all cases. Tree genotypes, however, significantly influenced peroxidase activity (Fig. 6).

In *Cinnamomum*, genotypic diversity level also exerted a greater effect than genotype in terms of its effect on most of the hydrolytic enzymes measured, the exception being NAG. However, the responses to genotypic diversity level for the two C acquisition enzymes differed from those of *Alniphyllum*. β-glucosidase and xylosidase activities were significantly higher in multi-genotypic than in mono-genotypic treatments (Fig. 5). Similar to the findings for *Alniphyllum*, acid phosphatase was higher in mono-genotypic than multi-genotypic treatments. Tree genotype, however, only significantly affected acid phosphatase activities (Fig. 5). The two oxidative enzymes measured were not affected by either genotypic diversity level or tree genotype.

In *Daphniphyllum*, tree genotype had a greater effect than tree genotypic diversity level on hydrolytic enzyme activities, in contrast to the findings for *Alniphyllum* and *Cinnamomum*. Xylosidase and acid phosphatase activities were significantly and solely influenced by tree genotype. On the other hand, phenol oxidase and peroxidase activities were mainly affected by tree genotypic diversity level; multi-genotypic treatment gave significantly lower enzyme activities in both cases (*P* < 0.01; Fig. 6).

## Discussion

Some recent studies have highlighted the effects of tree species and tree genotypes in shaping the associated soil microbial communities^1^,^35^. These results indicate the importance of inter- and intraspecific variations among host species in defining the structure of the microbial community in the soil. To complement these studies, we investigated the effects of tree species and tree genotype on soil enzyme activities, which are among the direct indicators of soil ecosystem functions. Since in real forest ecosystems different tree genotypes co-occur and interact, we also investigated the effects of genotypic diversity level on microbe-mediated soil ecosystem functions^36^. Our results highlight the importance of inter- and intra-specific variation, and especially of tree genotypic diversity, in shaping soil microbe-driven ecosystem functions across the subtropical experimental forests analyzed here.

We found a major effect of tree species identity (interspecific variation) on the overall, and most of the specific, patterns of soil enzyme activity. This is consistent with a recent study on the effects of tree species in a tropical montane forest^16^, which reported effects on overall soil enzyme activities and on two of the three specific enzymes (acid phosphatase and β-D-glucosidase) measured, mediated through effects on soil physicochemical and microbial properties. Similarly we also found that the most important factors affecting overall soil enzyme activities were tree species identity, followed by soil physicochemical properties (pH and water content). In total, tree species identity in combination with soil water content, pH and the shared fraction between tree species identity and pH and tree species identity and water content, explain 93% of the explainable variances. This finding may reflect the fact that in forest ecosystems, tree species can affect soil physicochemical properties and substrate quality by altering the quantity and quality of leaf litter input and also via root activities^15^,^16^,^17^.

The four tree species that we studied comprised both evergreen (*Cinnamomum* and *Daphniphyllum*) and deciduous (*Alniphyllum* and *Idesia*) species, thus the quantities and patterns of leaf litter fall varied greatly within this experimental design^37^. The physical (i.e. toughness) and chemical (C, N and phenolics and tannin concentrations) traits shown by leaf litter from these species also differed greatly^38^. *Alniphyllum fortunei* exhibits low leaf toughness with very high phenolic and tannin contents, whereas *Daphniphyllum* and *Cinnamomum* have tougher leaves with low levels of phenolics and tannins (Table S3). Litter quality, especially tannin concentration, has been reported to be responsible for differences in both soil microbial community and enzyme activities^16^. Specifically, *Dacrydium* (high tannin content) and *Lithocarpus* (low tannin content) exhibited significantly different overall enzyme activity patterns, and the soil under *Dacrydium* also had significantly higher acid phosphatase and β-D-glucosidase activities. Our results support the conclusion that tannin concentrations in leaf litter may affect soil enzyme activity underneath the tree, as *Alniphyllum fortunei* (high tannin) showed differences in overall enzyme activity that were significant compared with *Daphniphyllum* and *Cinnamomum* (both low-tannin species) respectively.

As in the case of species identity effects, the effects of intraspecific variations among tree genotypes on soil enzyme activities may be explained partly by genotype-specific influences on soil physicochemical properties and substrate quality. Particularly in the cases of *Alniphyllum*, *Cinnamomum* and *Idesia* tree genotypes and/or tree genotypic diversity were influential, along with soil physicochemical properties and/or substrate quality, in shaping overall enzyme activity patterns. Different tree genotypes can affect soil physicochemical properties and substrate quality by exerting different specific effects on litter chemical composition and root morphology, which subsequently affect soil microbial community structure^35^,^39^,^40^,^41^ and lead to unique soil microbial communities^35^. Interestingly, in *Daphniphyllum*, we found that genotypic diversity affected the overall soil enzyme activity pattern independently of the effects of the soil physicochemical properties and substrate quality that we measured. Specific enzyme activities were also affected by either tree genotype or tree genotypic diversity. Our results may thus indicate the existence of different host species-related drivers and mechanisms, and the findings highlight the effects of tree genotype and genotypic diversity in shaping soil enzyme activity patterns. Furthermore, the degrees of how different tree genotypes perform and adapt to the environmental parameters (including their feedback on the belowground microbial community) are not equal across different tree species, which might explain why we detected the effects of tree genotype or tree genotypic diversity level on soil enzyme activity patterns only in some cases (Table S2). Functional redundancy within microbial community may also explain our results as the microbial communities under different tree genotypes may be different but composed of communities that could produce similar enzymes^9^,^10^. In this study the level of genotypic diversity was found to be one of the most important predictors of overall soil enzyme pattern. This was also the case when each tree species was considered separately, as genotypic diversity level was significantly correlated with overall enzyme patterns in all tree species used in this experiment. In *Alniphyllum*, the activities of almost all specific soil enzymes were significantly higher in the mono-genotypic treatment than in multi-genotypic treatments. Similar results were found for acid phosphatase in the case of *Cinnamomum camphora*, and for *Daphniphyllum oldhamii* for all oxidative enzymes. This may have resulted from the combined effects of plant-microbe and microbe-microbe interactions as we have hypothesized^35^. As individual tree genotypes may be associated with unique soil microbial communities, competition among different soil microbial communities may be stronger under multi-genotypic treatment than under mono-genotypic treatment^1^,^35^. Where there is competition, high energy and resource use can directly and negatively impact the production of microbial enzymes^42^,^43^, resulting in the pattern that we observed.

In summary, we provide empirical evidence indicating that tree species identity and tree genotypes (inter- and intra-specific variation), as well as genotypic diversity levels, significantly influence soil ecosystem functions derived from microbial communities. These three factors influence the pattern of soil enzyme activity partly through their effects on soil physicochemical properties and substrate quality. In this ecosystem, both plant and soil related factors influence the pattern of soil microbe derived enzymes: tree species identity >soil pH >shared fractions [(between tree species identity and soil pH) and (tree species identity and soil water content)] >genotypic diversity level >soil water content. Furthermore, our results support the importance of intraspecific interaction (i.e. competition among different tree genotypes of a single tree species) in shaping the overall pattern of microbial enzymes in soils. An investigation of the soil and root-associated microbial communities of these tree species and tree genotypes across genotypic diversity levels using next generation sequencing tools is needed in order to provide a taxonomic and mechanistic understanding of the ways in which these factors affect plant-microbe and microbe-microbe interactions, and therefore microbially-mediated soil ecosystem functions (e.g. nutrient cycling, soil C pools) and developing forest management strategies in subtropical forests.

## Methods

### Study site

This experiment was conducted at the Biodiversity-Ecosystem Functioning (BEF)-China experimental platform located near Xingangshan village, Jiangxi Province (29.08–29.11 N, 117.90–117.93 E), P.R. China^34^,^44^. The climate of the study area is characterized as subtropical summer monsoon. The annual mean temperature and precipitation are 17.4 °C and 1635 mm, respectively. The experimental site was previously used as a *Cunninghamia lanceolata* plantation. In 2008, this plantation was clear-cut and the BEF-China experimental platform was established. Trees were planted in plots, with 400 tree individuals per plot (25.8 m × 25.8 m; planted in 20 rows of 20 tree individuals each), at a planting distance of 1.29 m, and the platform included a total of 24 tree species on 261 plots in order to investigate biodiversity effects on ecosystem functions^34^. Within this framework, the genetic diversity experiment represents a factorial combination of species diversity (1 or 4 species) and genetic diversity (1 or 4 maternal seed families per species) to specifically address the effects of genetic identity and genetic diversity. Henceforth we refer to a maternal seed family as a “genotype”, since members of a seed family share one parent in common and are on average more closely related than different seed families. A detailed description of the genetic diversity experiment has been given elsewhere^34^,^45^. Four tree species (*Alniphyllum fortunei* (Hemsley) Makino*, Cinnamomum camphora* (L.) Presl. *Daphniphyllum oldhamii* (Hemsley) Rosenthal and *Idesia polycarpa* Maximowicz.) were used in this experiment. For the present study, we used a subset of the genetic diversity experimental plots and investigated species-monocultures of the four species with either one (mono-genotypic treatment) or four (multi-genotypic treatment) seed families per species. Due to the large number of available monocultures of species and seed families, we used ¼ plots of 10 × 10 individuals for monocultures. No specific permissions were required for these locations and activities. The field studies did not involve endangered or protected species.

### Experimental design and sampling

Soil sampling was carried out in October 2014. Soil in the root zones of individual trees of four tree species, with four genotypes and at two genotypic diversity levels per species with four replicates each, was collected using an auger of diameter 8 cm and depth 10 cm. All soil samples were collected within one week in order to minimize the impact of weather variations. All plots were located at the same study site (site B), thus minimizing variation in soil physicochemical properties and quality. Within each plot we selected trees at minimum distances of 3 to 5 m apart to ensure independence among different soil samples. This distance is much larger than the spatial autocorrelation of soil enzymes measured in this study, which is within a range of a few square centimeters^13^ or up to 1 to 2 m^46^. Four soil cores were collected in four directions at 0.50–0.60 m from the base of each selected tree within the canopy projection area and pooled to produce one sample. We followed the roots from the tree base to ensure that we obtained soil samples influenced by the selected trees. The pooled samples were immediately homogenized and sieved (2 mm) in the field to remove stones, roots, macrofauna, and litter. Between samples the sieve was cleaned with a brush, sprayed with 70% ethanol and dried. After sieving, two subsamples (30 g) were immediately frozen in dry ice and transported to a laboratory near the study site. One subsample was stored at −20 °C until required for transport in dry ice to Germany for enzyme analysis. The other subsample was freeze-dried for soil physicochemical analyses. Further details of sampling design, characteristics of selected trees and soil physicochemical and substrate quality are shown in supporting information (Tables S1 and S2).

### Enzyme activities

Hydrolytic enzyme analyses were done for β-glucosidase (EC 3.2.1.21), xylosidase (EC 3.2.1.37), N-acetylglucosaminidase (EC 3.1.6.1) and phosphatase (EC 3.1.3.2) using 4-methylumbelliferone (MUB) as substrate as described elsewhere^27^,^47^. Briefly, soil slurries (sample suspensions) were prepared by adding 0.5 g soil to 50 ml of 50 mM sodium acetate buffer (pH 5.5) followed by homogenization for 5 min in a bath sonicator. Substrate solutions were prepared as follows: 150 (4-MUB-β-glucopyranoside, 4-MUB-β-D-xyloside and 4-MUB-phosphate) or 200 (4-MUB-N-acetyl-β-glucosaminide) μmol/L. Substrate blank wells received 50 μl substrate solution and 200 μl acetate buffer. Assay wells received 50 μl substrate solution and 200 μl soil slurry. Quench coefficient and emission coefficient wells received 50 μl of a MUB dilution series (concentrations (μmol/L) were: 2.5, 1.25, 0.625, 0.16) and 200 μl soil slurry or 200 μl acetate buffer. Homogenate blanks received 200 μl soil slurry and 50 μl acetate buffer. Plate blanks received 250 μl acetate buffer. There were eight replicate wells for each substrate blank, assay, quench standard, emission standard, homogenate blank and plate blank. The microplates were incubated in the dark at room temperature for 60 min. To stop the reaction, a 10 μl aliquot of 1.0 M NaOH was added to each well approximately 1 minute before reading the plates. Fluorescence was measured using a microplate fluorometer with 360 nm excitation and 465 nm emission filters. After correcting for blanks, emission and quenching, activities were expressed in units of nmol hr^−1^ g dry soil^−1^. Oxidative enzyme analyses were done for phenol oxidase (EC 1.10.3.2) and general peroxidase (EC 1.11.1.7) using 3, 3′, 5, 5′-tetramethylbenzidine (TMB) as substrate^48^. The protocol was slightly modified after pre-testing^27^. Briefly, sample suspensions were prepared as described above. Substrate solutions (TMB) were prepared in sodium citrate buffer with (peroxidase) or without (phenol oxidase) hydrogen peroxide at a concentration of 0.283 μmol/L. Substrate blank wells received 50 μl substrate solution and 200 μl 50 mM sodium acetate buffer. Assay wells received 50 μl substrate solution and 200 μl soil suspension. Homogenate blanks received 200 μl soil suspension and 50 μl sodium acetate buffer. Plate blanks received 250 μl sodium acetate buffer. This assay was also done with eight replicate wells. The microplates were incubated in the dark at room temperature for 20 min. To stop the reaction, a 30 μl aliquot of 1.5 M sulfuric acid was added to each well, and then plates were centrifuged at 2000 rpm for 5 min. Supernatant (200 μl) from each well (avoiding soil particles) was pipetted into a clean clear plate. The absorbance was measured using a microplate fluorometer with 260 nm excitation and 465 nm emission filters.

### Soil physicochemical property and quality analyses

Water content was calculated as the difference between initial and freeze-dried soil weight. Total C and N and C: N ratio were determined by dry combustion at 1000 °C with an Elementar Vario EL III (Hanau, Germany) elemental analyzer (DIN/ISO 10694 (Aug. 1996)). Soil pH was determined based on 10 g homogenized soil. Water content and pH were classed as soil physicochemical properties. The C: N ratio was used as a soil quality factor.

### Statistical analysis

The effects of tree species identity, tree genotype and genotypic diversity level on overall soil enzyme patterns for the six enzymes were analyzed using Permutational Multivariate Analysis of Variance (PERMANOVA) based on Euclidean distances implemented in PAST^49^. Statistical significances were based on 999 permutations and Bonferroni-corrected *P* values were applied in all cases when more than two groups were compared with PERMANOVA. Non-metric multidimensional scaling (NMDS) ordination based on Euclidean distances with the envfit function of the vegan package in R was used to investigate and visualize correlations among different factors influencing overall soil enzyme activities^50^. All significant factors (*P* < 0.05) were plotted in the respective NMDS ordinations. We determine the individual factors that were most influential for the overall pattern of soil enzyme activity using distance-based redundancy analysis (dbRDA), based on Euclidean and Bray–Curtis distances with the function capscale in vegan^51^. We tested for collinearity between all factors using Spearman’s rank correlation (ρ) before carrying out dbRDA. When two or more factors were correlated (ρ > 0.70 or ρ < −0.70 and *P* < 0.01), only one factor was retained for further analysis. In this case, only total soil C and total soil N were highly correlated (ρ = 0.86, *P* < 0.01), thus only N was kept for dbRDA. Four factors including tree species identity, genotypic diversity level, soil pH and soil water content were significant based on dbRDA and were selected for variance partitioning analysis. Variance partitioning analysis was performed using the varpart function in the vegan package in R to take into account the influence of four significant factors based on dbRDA (including both soil and plant related factors) on overall enzyme activity pattern^51^,^52^,^53^. We used the same enzyme activity matrix and factors as used for NMDS analysis. We also test whether shared fraction of these four factors explains the variance of soil enzyme activity^53^,^54^,^55^. The effects of tree species identity on specific enzyme activities were analyzed (as between plot factor) using split-plot ANOVA simultaneously accounting for tree genotype (within plot factor) and genotypic diversity level (between plot factor) (IBM SPSS Statistics 22, New York, NY, USA). Effects of tree genotype (within plot factor) and genotypic diversity level (between plot factors) were also analyzed for each tree species using a split-plot ANOVA. All data sets were tested for normality and the equality of group variances using a JB test and the Levene statistic^56^ and log10 transformed when necessary. Fisher’s Least Significant Difference (LSD) post hoc test was applied when more than two treatments were tested (IBM SPSS Statistics 22, New York, NY, USA).

## Additional Information

**How to cite this article**: Purahong, W. *et al*. Tree species, tree genotypes and tree genotypic diversity levels affect microbe-mediated soil ecosystem functions in a subtropical forest. *Sci. Rep.* **6**, 36672; doi: 10.1038/srep36672 (2016).

**Publisher’s note:** Springer Nature remains neutral with regard to jurisdictional claims in published maps and institutional affiliations.

## Supplementary Material

Supplementary Information

## Figures and Tables

**Table 1:** Goodness-of-fit statistics (*R* ^2^) for factors fitted to the non-metric multidimensional scaling (NMDS) ordination of overall patterns of enzyme activities.

Factor	All		*Alniphyllum*		*Cinnamomum*		*Daphniphyllum*		*Idesia*	
	*R*^2^	*P*	*R*^2^	*P*	*R*^2^	*P*	*R*^2^	*P*	*R*^2^	*P*
Tree species	0.29	**0.001**	nd	nd	nd	nd	nd	nd	nd	nd
Genotype	0.40	**0.001**	0.22	**0.030**	0.11	0.351	0.06	0.686	0.18	*0.099*
Genotypic diversity	0.07	**0.006**	0.21	**0.031**	0.29	**0.005**	0.19	**0.044**	0.20	**0.038**
pH	0.30	**0.001**	0.27	**0.011**	0.21	**0.034**	0.11	0.188	0.57	**0.001**
N	0.00	0.857	0.25	**0.011**	0.01	0.792	0.16	*0.070*	0.05	0.484
C	0.02	0.243	0.35	**0.003**	0.07	0.328	0.12	0.163	0.07	0.367
C: N	0.17	**0.001**	0.29	**0.003**	0.28	**0.013**	0.06	0.462	0.04	0.552
Water content	0.15	**0.001**	0.38	**0.002**	0.26	**0.019**	0.09	0.268	0.07	0.350

The significance was based on 999 permutations. Significant factors (*P* < 0.05) are indicated in bold. Marginally significant (*P* < 0.1) values are indicated in italics. nd = not determined.

**Table 2:** Significant predictors in the distance-based redundancy analysis (dbRDA) model of the overall pattern of soil enzyme activities.

Predictors	Euclidean distance		Bray–Curtis distance		
	*F*_model_	*P*	*F*_model_	*P*	
*Plant related factor*					
Tree genotypic diversity level	10.4117	**0.003**	6.2338	**0.001**	
Tree species identity	2.5580	**0.043**	4.4078	**0.001**	
*Soil related factor*					
Soil water content	9.3492	**0.002**	5.2925	**0.002**	
Soil pH	9.3492	**0.022**	4.4756	**0.007**	

P-values are based on 999 permutations.
