# Supplementary material for: Tree species, tree genotypes and tree genotypic diversity levels affect microbe-mediated soil ecosystem functions in a subtropical forest
Source: Sci Rep. 2016 Nov 18;6:36672. doi: 10.1038/srep36672 (PMC5114573; doi:10.1038/srep36672)
Supplement: Supplementary Information [file srep36672-s1.pdf]

Supplementary Information

Tree species, tree genotypes and tree genotypic diversity levels affect microbe-mediated soil ecosystem functions in a subtropical forest

Witoon Purahong[1], Walter Durka[2, 3], Markus Fischer[4], Sven Dommert[1], Ricardo Schöps[1], François Buscot[1, 3], Tesfaye Wubet[1, 3]

Affiliations:

[1]UFZ-Helmholtz Centre for Environmental Research, Department of Soil Ecology,Theodor-Lieser-Str. 4, D-06120 Halle (Saale), Germany;

[2]UFZ-Helmholtz Centre for Environmental Research, Department of Community Ecology, Theodor-Lieser-Str. 4, D-06120 Halle (Saale), Germany;

[3]German Centre for Integrative Biodiversity Research (iDiv), Halle-Jena-Leipzig, Deutscher Platz 5e, D-04103 Leipzig, Germany;

[4]Institute of Plant Sciences, University of Bern, Bern, Switzerland.

Table S1. Experimental design.

Tree Species (4)	Tree genotype (Seed family) (4)	Genetic diversity level (2)	Replicates (4)
Alniphyllum fortunei	DS2, DS101, DS104, DS108,	Monogenotypic (1 genotype) plot *vs.* multigenotypic (4 genotypes) plot	4
Cinnamomum camphora	D1, D2, D3, D6	Monogenotypic (1 genotype) plot *vs.* multigenotypic (4 genotypes) plot	4
Daphniphyllum oldhamii	DS5, DS18, DS19, DS21	Monogenotypic (1 genotype) plot *vs.* multigenotypic (4 genotypes) plot	4
Idesia polycarpa	D3, D5, D10, D12	Monogenotypic (1 genotype) plot *vs.* multigenotypic (4 genotypes) plot	4

Table S2. Characteristics of selected trees and soil physicochemical and substrate quality.

Tree species	Seed family	Genetic Diversity level	Mean tree height (cm)	pH	Total N (%)	Total C (%)	C:N
Alniphyllum fortunei	DS101	1	135	3.77	0.18	2.16	12.18
Alniphyllum fortunei	DS101	4	153	3.76	0.18	2.55	14.38
Alniphyllum fortunei	DS104	1	105.5	3.67	0.21	2.86	13.69
Alniphyllum fortunei	DS104	4	135.5	3.78	0.17	2.65	15.30
Alniphyllum fortunei	DS108	1	121.25	3.91	0.20	2.84	14.35
Alniphyllum fortunei	DS108	4	187.5	3.69	0.19	3.18	16.45
Alniphyllum fortunei	DS2	1	120.75	3.71	0.15	1.70	11.31
Alniphyllum fortunei	DS2	4	129.75	3.71	0.18	2.97	16.56
Cinnamomum camphora	D1	1	73	3.98	0.18	2.36	13.32
Cinnamomum camphora	D1	4	59	3.86	0.18	2.71	14.78
Cinnamomum camphora	D2	1	79.5	3.91	0.18	2.36	13.47
Cinnamomum camphora	D2	4	62	3.9	0.17	2.44	14.76
Cinnamomum camphora	D3	1	79	3.86	0.18	2.70	15.07
Cinnamomum camphora	D3	4	63.25	3.78	0.20	3.21	15.98
Cinnamomum camphora	D6	1	72.5	3.78	0.16	2.33	15.06
Cinnamomum camphora	D6	4	67.5	3.8	0.22	3.26	14.88
Daphniphyllum oldhamii	DS18	1	115.25	3.98	0.27	3.87	14.28
Daphniphyllum oldhamii	DS18	4	107	3.95	0.16	2.38	14.50
Daphniphyllum oldhamii	DS19	1	111.75	3.94	0.15	2.20	14.99
Daphniphyllum oldhamii	DS19	4	134.75	3.96	0.12	1.54	12.55
Daphniphyllum oldhamii	DS21	1	112.25	3.86	0.18	2.36	13.49
Daphniphyllum oldhamii	DS21	4	116.75	3.95	0.14	1.87	13.64
Daphniphyllum oldhamii	DS5	1	84	4	0.28	3.64	13.16
Daphniphyllum oldhamii	DS5	4	121.25	3.98	0.17	2.55	14.75
Idesia polycarpa	D10	1	81.25	3.87	0.19	2.11	11.35
Idesia polycarpa	D10	4	130.5	3.84	0.16	2.25	13.78
Idesia polycarpa	D12	1	169	3.85	0.15	1.70	11.58
Idesia polycarpa	D12	4	74	3.94	0.18	2.56	13.90
Idesia polycarpa	D3	1	124.25	3.83	0.21	3.01	14.28
Idesia polycarpa	D3	4	77.75	3.95	0.21	2.91	14.19
Idesia polycarpa	D5	1	93.5	3.69	0.19	2.60	13.47
Idesia polycarpa	D5	4	62.25	3.86	0.14	2.04	14.14

Table S3. Leaf toughness, total phenolics concentration and tannin concentration of tree species used in this experiment.

Species	Leaf toughness Mean (range)	Total phenolics concentration Mean (range)	Tannin concentration Mean (range)	References
Alniphyllum fortunei	0.20 (0.16 – 0.27)	163.90 (139.57 – 194.50)	117.07 (107.21 – 132.84)	Eichenberg et al., 2015
Cinnamomum camphora	0.60 (0.46 – 0.74)	25.63 (13.43 - 43.98)	20.84 (13.76 – 28.29)	Eichenberg et al., 2015
Daphniphyllum oldhamii	0.54 (0.39 – 0.70)	19.86 (11.12 – 25.86)	9.70 (6.07 – 13.16)	Eichenberg et al., 2015
Idesia polycarpa	n.d.	n.d.	n.d.	

Reference

Eichenberg D, Purschke O, Ristok C, Wessjohann L, Bruelheide H (2015) Trade-offs between physical and chemical carbon-based leaf defence: of intraspecific variation and trait evolution. J Ecol 103:1667–1679.
